# Adhesion of the genome-sequenced *Lactococcus lactis* subsp. *cremoris* IBB477 strain is mediated by specific molecular determinants

**DOI:** 10.1007/s00253-016-7813-0

**Published:** 2016-09-29

**Authors:** Joanna Maria Radziwill-Bienkowska, Doan Thanh Lam Le, Pawel Szczesny, Marie-Pierre Duviau, Tamara Aleksandrzak-Piekarczyk, Pascal Loubière, Muriel Mercier-Bonin, Jacek Karol Bardowski, Magdalena Kowalczyk

**Affiliations:** 1 Institute of Biochemistry and Biophysics, Polish Academy of Sciences, Pawinskiego 5A, 02-106 Warsaw, Poland; 2 LISBP - Université de Toulouse, CNRS, INRA, INSA, Toulouse, France; 3 Faculty of Biology, University of Warsaw, Pawinskiego 5A, 02-106 Warsaw, Poland; 4 INRA/INPT/UPS Toxalim UMR 1331, 180 chemin de Tournefeuille, F-31027 Toulouse, France

**Keywords:** *Lactococcus lactis*, Adhesive properties, Mucin, Shear stress flow chamber, Genome sequence, Adhesins

## Abstract

Understanding the nature of mucus-microbe interactions will provide important information that can help to elucidate the mechanisms underlying probiotic adhesion. This study focused on the adhesive properties of the *Lactococcus lactis* subsp. *cremoris* IBB477 strain, previously shown to persist in the gastrointestinal tract of germ-free rats. The shear flow-induced detachment of *L. lactis* cells was investigated under laminar flow conditions. Such a dynamic approach demonstrated increased adhesion to bare and mucin-coated polystyrene for IBB477, compared to that observed for the MG1820 control strain. To identify potential genetic determinants giving adhesive properties to IBB477, the improved high-quality draft genome sequence comprising chromosome and five plasmids was obtained and analysed. The number of putative adhesion proteins was determined on the basis of surface/extracellular localisation and/or the presence of adhesion domains. To identify proteins essential for the IBB477 specific adhesion property, nine deletion mutants in chromosomal genes have been constructed and analysed using adhesion tests on bare polystyrene as well as mucin-, fibronectin- or collagen IV-coated polystyrene plates in comparison to the wild-type strain. These experiments demonstrated that gene AJ89_07570 encoding a protein containing DUF285, MucBP and four Big_3 domains is involved in adhesion to bare and mucin-coated polystyrene. To summarise, in the present work, we characterised the adhesion of IBB477 under laminar flow conditions; identified the putative adherence factors present in IBB477, which is the first *L. lactis* strain exhibiting adhesive and mucoadhesive properties to be sequenced and demonstrated that one of the proteins containing adhesion domains contributes to adhesion.

**Electronic supplementary material:**

The online version of this article (doi:10.1007/s00253-016-7813-0) contains supplementary material, which is available to authorized users.

## Introduction

The mucosal epithelium of the gastrointestinal tract (GIT) displays highly specialised functions, like the digestion and absorption of ingested food and elimination of undigested food, microorganisms, and microbial products. To protect the mucosa, the host produces a layer of mucus covering the stomach, small intestine and large bowel (Atuma et al. 2001; Cone 2009). This protective barrier, which constitutes the first line of defence against physical and chemical injury (Neutra and Forstner 1987), consists of two adjacent layers: a thin inner layer, which is sterile and physically difficult to dislodge, and a thicker outer one, which is not sterile and more diffuse (Johansson et al. 2008, 2011). The major components of mucus are mucins, which are responsible for its viscoelastic gel-like and biological properties. The membrane-bound and secreted mucins are large glycoproteins, with a protein backbone linked to a complex array of hydrophilic oligosaccharide side chains (Bansil and Turner 2006), which represent potential ligands for microbial adhesins and/or an energy source for microorganisms in the outer mucus layer (McGuckin et al. 2011). Biological and physical interactions with the mucus layer, and especially mucins, are increasingly identified as an important trait in improving the gut ecology through a proper balance of putative beneficial bacteria, like probiotic lactic acid bacteria (LAB), over pathogens. Understanding the nature of mucus-microbe interactions would be an important step in elucidating the mechanisms of probiotic adhesion. In vitro and in vivo studies on bacterial adhesion have been broadly performed for lactobacilli (Van Tassell and Miller 2011), but they are limited in other LAB including streptococci (Kebouchi et al. 2016) and lactococci (Le et al. 2013).

*Lactococcus lactis*, considered as the model LAB, is widely used as a starter in manufacturing cheese and other fermented dairy products. Although lactococci are not a frequent natural element of the intestinal microbiota, they can be used in food (probiotics) and health-related applications (mucosal delivery systems). Adhesive properties can prolong the contact between bacteria and the host and therefore enhance the desired probiotic effect and promote a protective immune response of mucosal vaccines. Some *L. lactis* strains were recently shown to survive for a long time in the GIT of rodents and to adhere to the intestinal mucosa (Boguslawska et al. 2009; McNulty et al. 2011; Wang et al. 2011).

In view of the above, the present work focused on the adhesive and mucoadhesive properties of *L. lactis* subsp. *cremoris* IBB477. This strain, originally isolated from Polish raw milk, was shown to be resistant to tetracycline and persistent in the GIT of germ-free rats (Boguslawska et al. 2009). IBB477 is a candidate strain for development of an oral protective vaccine against avian influenza virus infections, based on the method used in preliminary experiments on *L. lactis* IL1403 (Szatraj et al. 2014). Adhesion of IBB477 cells to an abiotic polystyrene surface, bare or coated with a model mucin (pig gastric mucin (PGM)), was quantified at the single-cell scale by atomic force microscopy (AFM) (Dague et al. 2010; Le et al. 2011) and further at the bacterial population level, by using quartz crystal microbalance with dissipation monitoring (Le et al. 2012) and using the microtiter plate method (Radziwill-Bienkowska et al. 2014). However, the cell surface components involved in the adhesion and mucoadhesion of IBB477 cells have remained unknown so far, except for the presence of *prtP* and *mub* genes in the genome, which code for serine proteinase and LPXTG-anchored mucus-binding protein containing MucBP domains (PF06458), respectively (Radziwill-Bienkowska et al. 2014). PrtP- and MucBP-domain-containing proteins have been shown to play a crucial role in bacterial adhesion to solid surfaces (Habimana et al. 2007) or to mucus (Van Tassell and Miller 2011).

Furthermore, because sessile microorganisms are often subjected to a flowing carrier fluid in many abiotic and biotic environments, like the GIT, it is important to unravel the relationship between bacterial mucoadhesion and shear flow susceptibility. In this framework, the interactions between *L. lactis* cells and bare or PGM-coated polystyrene were probed under dynamic conditions, by using the shear stress flow chamber. Shear flow-induced detachment experiments were carried out under well-controlled laminar flow, with IBB477 and the low-adhesive MG1820, as the control. In addition, to support the results of detachment experiments, putative genetic determinants encoding the adhesive capacity of IBB477 were identified by genome sequencing and bioinformatics analysis. Finally, the role in the adhesion of several proteins containing adhesion domains was verified in tests on polystyrene as well as mucin-, fibronectin- or collagen IV-coated polystyrene.

## Materials and methods

### Bacterial strains and growth conditions

The following strains were used in this study: wild-type *L. lactis* subsp. *cremoris* MG1820 (Maeda and Gasson 1986), (LISBP—Université de Toulouse, CNRS, INRA, INSA, Toulouse, France), *L. lactis* subsp. *lactis* IL1403 (Chopin et al. 1984), (INRA, Jouy-en-Josas, France), *L. lactis* subsp. *cremoris* IBB477 (IBB PAS, Warsaw, Poland; deposited in the Polish Collection of Microorganisms (PCM) culture collection no. 2853), *Escherichia coli* TG1 [Δ(*hsdMS-mcrB*) 5 Δ(*lac-proAB*) *supE thi-1*F’(*traD36 proAB+ lacI* ^q^ *Z*Δ*M15*)] (laboratory collection) and *E. coli* EC1000 (Km^r^, RepA^+^ MC1000, carrying a single copy of the pWV01 *repA* gene in the *glgB* gene) (laboratory collection). The IBB477 strain was originally isolated from samples of Polish raw milk (Boguslawska et al. 2009). The IBB477 deletion mutants created in this study are listed in Table 1. The wild-type IBB477 strain, which is tetracycline resistant, and its mutants were grown on the medium supplemented with 10 μg ml^−1^ of tetracycline (Sigma-Aldrich, Inc., St. Louis, MO, USA). Lactococcal strains were generally cultured on M17-glucose (0.5 % *w*/*v*) at 30 °C (M17; Oxoid Ltd., Basingstoke, Hampshire, UK), except for bacteria detachment experiments in the shear stress flow chamber (see below). In this case, strains were cultured in M17-lactose (2 % w/v) medium. *Lactococcus* stock cultures were kept at −80 °C in M17 broth, containing 20 % (*v*/*v*) glycerol and 0.5 % (*w*/*v*) glucose or 2 % (*w*/*v*) lactose, as indicated above. *E. coli* cells were grown in Luria-Bertani (LB) medium at 37 °C. Selection of pGEM-T Easy constructs in *E. coli* TG1 was performed on selective LB medium (60 μg ml^−1^ X-Gal; 0.3 mM IPTG; 50 μg ml^−1^ ampicillin). For construction of deletion mutants using the thermosensitive plasmid pGhost9 (Maguin et al. 1996), erythromycin (Em; 100 μg ml^−1^ for *E. coli* and 5 μg ml^−1^ for *L. lactis*) was added to the medium and specific temperatures were applied as described below.

**Table 1:** The IBB477 deletion mutants created in this study and primer pairs used for their construction

Mutant’s name^a^	IBB PAS culture collection no.	GenBank locus_tag	Primers
Δcna1	3180	AJ89_05215 and AJ89_05220	cna1-u_F AATACGGCAACCCTTTATCC cna1-u_R CGAATTC-TGCAACGGTACCCGTTTCTG (*Eco*RI) cna1-d_F CGAAT-TCTTGGTGTTGCCATGGTTG (*Eco*RI) cna1-d_R CCACTTGGTAGGCATGTATC
Δfbp1	3181	AJ89_06570	fbp1-u_F TCCACCTGTTCCATTTAC fbp1-u_R GCGGATCC-CAAGTGCTTGCTAAAGTC (*Bam*HI) fbp1-d_F GCGGATCC-AAGAAATGATGGCAGCCAGAC (*Bam*HI) fbp1-d_R CCGAGCAATGCATGAATCTCAC
Δcna2	3182	AJ89_06625 and AJ89_06630	cna2-u_F CACAAGTGAGAGCCAGAATG cna2-u_R CGAATTC-ATCTGGGTTGCTACCATTG (*Eco*RI) cna2-d_F CGAATTC-CATAAAACTGGGTGAATCC (*Eco*RI) cna2-d_R TTTACCTCCGATTGGATATG
Δbig	3183	AJ89_07570	big-u_F GCCAAGTATAGGCATTTATAAG big-u_R CGAATTC-TCGGTGAAAGAGTCCCAAGTAAC (*Eco*RI) big-d_F CGAATTC-TTTGATTTCGGTCTTGATGG (*Eco*RI) big-d_R TGCGAGCAGTGTCATGTTTC
Δfbp2	3184	AJ89_09345	fbp2-u_F CGACATCGTTGAAGGTACAG fbp2-u_R CGAATTC-TTGCCAGATTTCCATGTTCG (*Eco*RI) fbp2-d_F CGAATT-CTCAAGCTATTGATGAAAG (*Eco*RI) fbp2-d_R TAGGTGTCAACAGTGTTTA
Δchw	3185	AJ89_10320	chw-u_F ACGAGAAGGCTACTCAGAAC chw-u_R CGAAT-TCTTGTTGCTCGACAGTAAATGC (*Eco*RI) chw-d_F CGAATT-CTCCATAAGTGTAAAATAAC (*Eco*RI) chw-d_R TTCTTAGACCAAGGTTCTTC
Δchit	3186	AJ89_11550	chit-u_F GGTTATGTGAAAGACCCTAATG chit-u_R CGAATT-CCAGCAGTAACTAATAAACC (*Eco*RI) chit-d_F CGAAT-TCTTTGGACCCTTCTTGTAAAC (*Eco*RI) chit-d_R TCTTGAACGATGCCCTTGATG
Δvwa	3187	AJ89_11995	vwa-u_F CAACCACACTCTGGTATTG vwa-u_R CGAATTC-ACCTTAGGTCCTTGCATTAG (*Eco*RI) *vwa-d_F GGAATTC-*TAGGTGGAATTGGTTTGACTATCG (*Eco*RI) vwa-d_R AGCCTAATGAATACTGGCTGTA
Δmub	3188	AJ89_12755	mub-u_F AGAGTCAAGTGCGATGGAAG mub-u_R GC*GGAT*-*CC*ACCAGCAAGAATCGTTAAGG (*Bam*HI) mub-d_F GCGGATCC-TCTGACAGGGTCAGGCTTT (*Bam*HI) mub-d_R GTGTGCGTTCGCCATAGAT

^a^Strains obtained in this study are deposited in the publicly accessible IBB PAS laboratory culture collection

### Preparation of mucins

The starting material, type III mucin from porcine stomach (PGM) (lyophilised powder, cat. no. M1778; Sigma-Aldrich, St. Louis, MO, USA), was dissolved in phosphate-buffered saline (PBS; pH = 7.5) to a concentration of 10 mg ml^−1^ just before use.

### Adhesion assay under dynamic conditions by using the shear stress flow chamber

Cell suspensions were prepared as follows: once the early stationary phase of growth was reached (optical density at 580 nm (OD_580nm_) of 5.0), the bacteria were harvested by centrifugation (2900×*g*, 10 min at room temperature) and washed twice with PBS. The OD_580nm_ of the suspension was then adjusted to 0.25, which approximately corresponds to 1.25 × 10^8^ colony-forming units (cfu) ml^−1^ (as determined by viable count method).

Polystyrene (Arias, Toulouse, France) was used in the form of rectangular coupons (25.2 mm × 6.3 mm × 2.0 mm). The experimental procedure, previously described for *L. lactis* (Le et al. 2013), was slightly modified. In brief, the detachment of *L. lactis* cells from bare or PGM-coated polystyrene coupons (PS and PS + PGM, respectively) was analyzed in a rectangular flow channel (12.0 mm wide, 25.2 mm long and 200 μm thick). The flow chamber and all tubes were first filled with PBS, ensuring that no air bubbles are trapped in the system. The cell suspension (700 μl, OD_580_ = 0.25) was then slowly injected into the flow chamber and the cells were allowed to attach to the solid surface (PS or PS + PGM) under static conditions for 3 h. Images, captured using the reflection mode of an optical microscope (Eclipse LV100; Nikon France Instruments, Champigny sur Marne, France) equipped with × 40 ultra-long working distance objective, were recorded by a camera (Digital STGHT DS-2MBW; Nikon France Instruments, Champigny sur Marne, France) and the NIS-Elements F3.0 video acquisition software. The field of view was 144 μm × 108 μm, with a resolution of 0.09 μm per pixel. Images were analyzed to estimate the percentage of the surface occupied by attached cells by using the free software Macbiophotonics ImageJ and the Matlab software (Mathworks Inc., USA).

After the 3-h adhesion step (initial surface coverage denoted as A_i_), PBS rinse was performed at a low flow rate of 0.002 ml s^−1^ to stabilise the system and to remove loosely attached bacteria. The percentage of the remaining attached cells is hereafter referred as to as A_0_. The A_0_ value was 1–3 % of the total surface area, so any interactions between neighbouring bacteria were considered as minimal. Laminar flow of PBS was then imposed, with a stepwise increase in the flow rate (maximal value of 6.7 ml s^−1^), with a duration of 3 min for each step. Flow rates ranging from 0.002 to 0.3 ml s^−1^ were generated by gravity and controlled through a toothed rack, which was of the height of a constant head vessel located upstream of the chamber. Higher flow rates were obtained using a gear pump (Ismatec; Fisher Bioblock Scientific, Illkirch, France). The wall shear stress τ_W_ is given by

$$ {\tau}_W=\frac{3\mu Q}{4{\displaystyle {h}^2}l} $$

where *μ* is the fluid dynamic viscosity (Pa s); *Q* (m^3^ s^1^) is the flow rate and *l* and *h* are, respectively, the channel half-width and half-thickness (m). The applied wall shear stress was in the range 0–80 Pa.

At the end of each step, the surface covered by attached bacteria (*A*) was estimated. The detachment profile, representing the ratio A/A_0_ as a function of the wall shear stress, was plotted. Each condition (PS vs. PS + PGM) was performed in triplicate for the IBB477 and MG1820 strains with different coupons and separately grown cultures.

### DNA sequencing, sequence analysis and assembly

The genomic DNA was extracted using the Genomic Maxi AX purification kit (A&A Biotechnology, Gdynia, Poland) preceded by incubation in TES-lysozyme (20 mg ml^−1^) for 30 min at 37 °C. The genome sequence of the *L. lactis* IBB477 strain was generated by shotgun and paired-end reads by using the GS FLX Titanium platform (F. Hoffmann-La Roche AG, Basel, Switzerland) and the Illumina sequencing technology. Sequence assembly was carried out using the Newbler Assembler version 2.4 software (F. Hoffmann-La Roche AG, Basel, Switzerland). The chromosome assembly was validated with MapSolver based on the optical map produced via OpGen’s Optical Mapping System for the whole genome of the IBB477 strain by using the restriction enzyme *Afl*II. Gap resolution within plasmids was performed by PCR and sequencing using sequence-specific primers. Automatic annotation of the genome was generated using the National Center for Biotechnology Information (NCBI) Prokaryotic Genome Annotation Pipeline (PGAP; http://www.ncbi.nlm.nih.gov/genome/annotation_prok/) version 2.0, released in May 2013, using the protein homology and GeneMarkS+ prediction program.

### Bioinformatics analysis

Sequence comparison of *L. lactis* IBB447 to other *L. lactis* subsp. *cremoris* strains was done with BRIG (Alikhan et al. 2011) and Mauve (Darling et al. 2010). Insertion elements were identified using ISfinder (Siguier 2006). Subcellular localisation of proteins was predicted using PSORTb version 3.0.2 (Yu et al. 2010). The putative adhesion domains in the encoded amino acid sequences were found using Pfam database version 27.0 (Finn et al. 2014).

### Construction of deletion mutants

Mutants were created by double crossover between pGhost9 harbouring DNA fragments flanking the deleted regions and the chromosomal region containing these DNA fragments. The flanking upstream and downstream DNA fragments (400–1000 bp) were amplified with ExTaq polymerase (TaKaRa Bio, Inc., Shiga, Japan) using the appropriate forward and reverse primer pairs (Table 1) containing *Eco*RI or *Bam*HI sites, enabling ligation of both fragments. The amplified PCR fragments after *Eco*RI or *Bam*HI digestion were cloned to the pGEM-T Easy vector (Promega, Madison, WI, USA) providing a compatible overhang for ligation of PCR products generated by thermostable polymerases that add a single deoxyadenosine to the 3′-ends of amplified fragments. Ligated fragments were subsequently reamplified with ExTaq polymerase using the forward primer of the upstream region and the reverse primer of the downstream region. PCR reaction was performed on the ligation mixture as a template or using colonies after introducing the generated construct into the *E. coli* TG1 cells. Each amplified region containing deletion was cloned to the modified pGhost9 vector with added 3′ terminal thymidine to both ends after *Eco*RV digestion. To prepare 1 μg of pGhost9 vector, 5 nmol of 2′,3′-dideoxythymidine-5′-triphosphate (ddTTP) (Affymetrix, Santa Clara, CA, USA), 90 U of terminal deoxynucleotidyl transferase (TdT) (Thermo Fisher Scientific, Waltham, MA, USA) and dedicated 1× reaction buffer for TdT were added. The reaction mixture of 60 μl was incubated at 37 °C for 1.5 h and subsequently subjected to enzyme inactivation by heating (70 °C for 10 min). This approach enabled direct cloning of PCR products into pGhost9 vector and, after selection of the proper construct in *E. coli* EC1000, its introduction into electrocompetent *L. lactis* IBB477 cells. Selected regions were deleted from the chromosome of IBB477 using an integration-excision system (Maguin et al. 1996) according to the following procedure. Strains containing constructed plasmids were grown at 28 °C overnight in the M17-glucose medium with tetracycline and erythromycin for plasmid selection. Homologous recombination was enforced by 100-fold dilution of the saturated lactococcal culture in M17-glucose medium with tetracycline and incubation for 2.5 h at 28 °C and 2.5 h at 37.5 °C. Integrants containing pGhost9 constructs in the chromosome were selected at 37 °C on M17-glucose agar plates containing tetracycline and erythromycin. Excision from the chromosome and removal of the integration vector from *L. lactis* were performed by growth of integrants in the absence of erythromycin. To this end, integrants were cultured overnight in M17-glucose medium with tetracycline at 37 °C. Cultures were then diluted 10^6^-fold in the fresh medium and incubated at 28 °C until saturation (about 18 h). Appropriate dilutions of the saturated cultures were incubated on plates without erythromycin selection at 37 °C to allow plasmid loss. Colonies were transferred with toothpicks to selective and non-selective plates to detect erythromycin sensitive cells, in which excision had occurred. The genetic structure of the resulting deletion strains was confirmed by colony PCR and sequencing of the DNA region containing the deleted gene.

### Complementation of the deleted gene

In order to complement the deleted gene encoding AJ89_07570 protein (Δbig), this gene with its putative promoter region was amplified with Phusion High-Fidelty DNA Polymerase (New England Biolabs, USA) using pbig_F (CGCCCGG-GTAGATTATCTCAAGGGTGGTTAG) and pbig_R (CCGCTCGAG-TAACGTTTGTTAAGTCTTTC) primers. The resulting fragment was cloned into pGhost9 using *Cfr9*I (*Xma*I) and *Xho*I restriction enzymes, transferred into *E. coli* EC1000 and, after selection of the proper construct, transferred into electrocompetent *L. lactis* IBB477 Δbig mutant, giving rise to the IBB477Δbig + pGhost9big strain (IBB PAS culture collection no. 3191).

### Preparation of mucin-, collagen- or fibronectin-coated polystyrene plates

Solutions o: (i) type III mucin from porcine stomach (PGM) (cat. no. M1778, Sigma-Aldrich, St. Louis, MO, USA) (10 mg ml^−1^), (ii) fibronectin from human plasma (FN) (cat. no. F2006, Sigma-Aldrich, St. Louis, MO, USA) (20 μg ml^−1^) and (iii) collagen from human placenta type IV (CN IV) (cat. no. C7521, Sigma-Aldrich, St. Louis, MO, USA) (20 μg ml^−1^) were all dissolved in phosphate-buffered saline (PBS), pH = 7.4 (BioShop Canada Inc., Burlington, Ontario, Canada) just before use. Adhesion of *L. lactis* to PGM, FN and CN IV was determined on polystyrene 96-well microtiter plates (cat. no. 167,008, Thermo Fischer Scientific Nunc A/S, Roskilde, Denmark) coated with 200 μl (PGM and CN) or 150 μl (FN) of the prepared solutions and incubated overnight at 4 °C, with gentle agitation on a platform rocker shaker. After incubation, the wells were washed three times with PBS and five times with sterile Milli-Q-grade water (PGM) or three times with PBS (FN and CN) to remove loosely bound material. The plates were air-dried and used directly after preparation.

### Adhesion tests

Adhesion of bacterial cells to bare polystyrene (PS), PGM-coated (PS + PGM), fibronectin-coated (PS + FN) or collagen IV-coated (PS + CN IV) polystyrene was tested on the 96-well microtiter plates (cat. no. 167008, Thermo Fischer Scientific Nunc A/S, Roskilde, Denmark), using the technique previously described for the IBB477 strain (Radziwill-Bienkowska et al. 2014) with slight modifications. Bacteria from overnight cultures diluted to OD_660 nm_ of 1.0 were harvested by centrifugation at 9000×*g* for 1 min and resuspended in an equal volume of PBS. A volume of 100 μl of bacterial suspension was added to each well (at least six for each strain). After 3-h incubation under static conditions at 30 °C, the wells were carefully washed two times with 300 μl and one time with 400 μl of sterile Milli-Q-grade water to remove unbound bacteria. Bound cells were stained with crystal violet (cat. no. 109218, Merck, Darmstadt, Germany) (100 μl per well) at room temperature for 10 min and rinsed three times with water as above to remove excess stain. Finally, stained bacteria were suspended in 200 μl of 96 % ethanol and optical density was determined at 583 nm (OD_583nm_) on a Synergy HT Multi-Detection Reader (BioTek Instruments Inc., Winooski, VT, USA). The average value of at least six measurements was calculated after rejecting extreme results. Bacterial adhesion was determined in three independent experiments, and the results are presented as means ± standard deviations. A statistical analysis was performed using Welch *t* test. Each microtiter plate included the control strains: *L. lactis* MG1820, *L. lactis* IL1403 and blank wells with PBS.

### Nucleotide sequence accession number

The genome studied under this Whole Genome Shotgun project has been deposited at DDBJ/EMBL/GenBank under the accession number JMMZ00000000. The version described in this paper is JMMZ01000000.

## Results

### Shear flow-induced detachment of *L. lactis* cells from bare and PGM-coated polystyrene: comparison between IBB477 and MG1820

The shear stress flow chamber (Guillemot et al. 2006; Mercier-Bonin et al. 2011) was used for monitoring the shear flow-induced detachment of IBB477 and MG1820 cells to compare their adhesive/mucoadhesive properties, probed under well-controlled laminar flow.

First, we focused on PBS rinse at low wall shear stress (τ_W_ = 0.03 Pa) after the 3-h adhesion phase under static conditions. Table 2 displays the values of A_0_/A_i_ ratio (with A_i_ and A_0_ being the surface coverage by cells before and after rinsing, respectively) for IBB477 and MG1820 cells attached to polystyrene without (PS) or with PGM coating (PS + PGM). It is noteworthy that for PS + PGM, whatever the strain be, the initial surface coverage A_i_ was lower than that obtained with PS (for instance, 2.4 ± 0.9 and 1.1 ± 0.8 % for MG1820 strain on PS and PS + PGM, respectively). Furthermore, the effect of PBS rinse was more pronounced in the case of PS + PGM (for instance, an A_0_/A_i_ ratio of 95.1 ± 4.0 and 67.9 ± 18.6 % for MG1820 strain on PS and PS + PGM, respectively). This indicates the antiadhesive effect of the adsorbed PGM on MG1820 cells, and to a lesser extent, IBB477 cells (67.9 ± 18.6 and 89.5 ± 10.3 % on PS + PGM for MG1820 and IBB477 strains, respectively).

**Table 2:** Effect of rinsing with phosphate-buffered saline (PBS) at wall shear stress (τ_W_ = 0.03 Pa), after the 3-h adhesion phase, on *L. lactis* cells (IBB477 and MG1820 strains) attached to bare (PS) or mucin-coated (PS + PGM) polystyrene surfaces

Surface	PS		PS + PGM	
Strain	MG1820	IBB477	MG1820	IBB477
A_i_, before PBS rinse (%)	2.4 ± 0.9	3.0 ± 0.4	1.1 ± 0.8	2.1 ± 0.7
A_0_, after PBS rinse (%)	2.3 ± 0.9	2.9 ± 0.4	0.7 ± 0.5	1.9 ± 0.7
A_0_/A_i_ (%)	95.1 ± 4.0	97.3 ± 1.5	67.9 ± 18.6	89.5 ± 10.3

The results presented are the average values and standard deviations for three different coupons and separately grown cultures

*A*_*0*_ surface covered by cells after rinsing, *A*_*i*_ surface covered by cells before rinsing

**Fig. 1:**
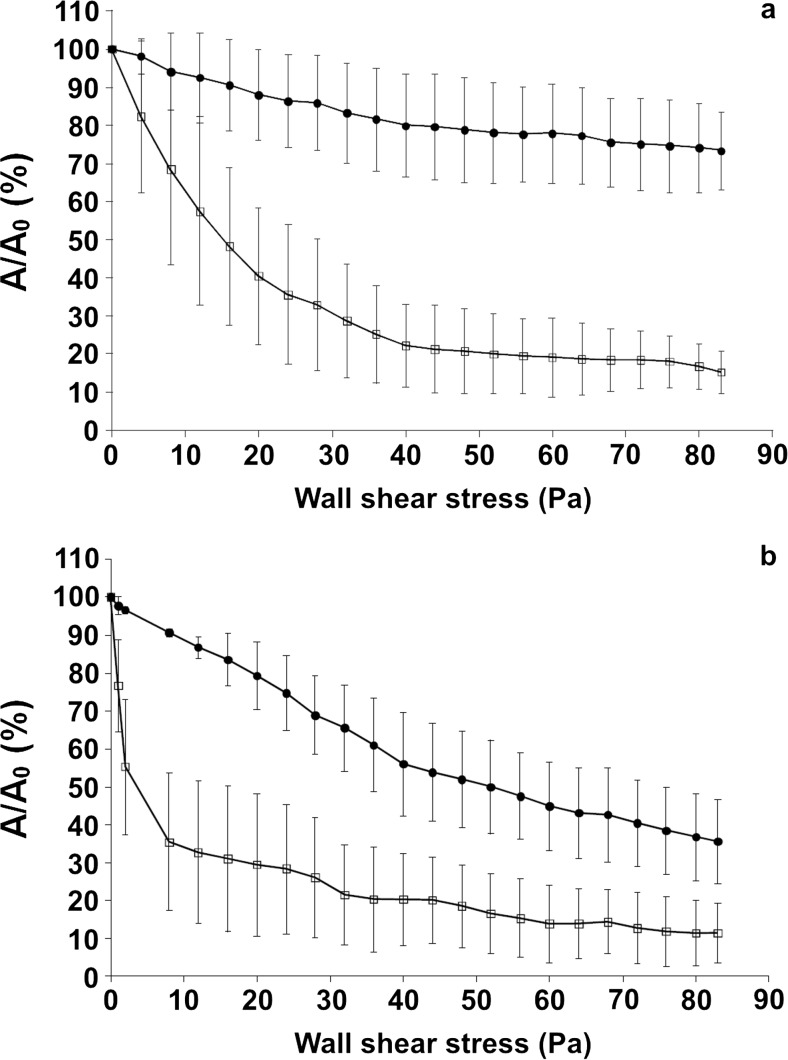
Shear flow-induced detachment profiles of *L. lactis* cells attached to **a** bare polystyrene (PS) and **b** PGM-coated polystyrene (PS + PGM) in PBS. (*Black circle*) IBB477 strain; (*white square*) MG1820 strain. The results presented are the average values and standard deviations for three different coupons and separately grown cultures

Subsequent detachment profiles for IBB477 and MG1820 strains are presented in Fig. 1a, b for PS and PS + PGM, respectively. Adhesion of the *L. lactis* cells to PS highly depended on the strain (Fig. 1a). For the MG1820 strain, increasing the wall shear stress progressively decreased the fraction of attached bacteria and a maximal detachment of 80 % was achieved. In contrast, for the IBB477 strain, the fraction of detached bacteria was significantly lower, and at the end of the experiment (τ_W_ = 80 Pa), around 75 % of the initial bacterial population remained attached to PS. After PGM coating, adhesion level was significantly lower for both strains (Fig. 1b). For the control MG1820 cells, adhesion to PS + PGM was markedly reduced, especially for low τ_W_ values. Adhesion of IBB477 cells to PS + PGM was significantly enhanced compared to MG1820; at the end of the experiment (τ_W_ = 80 Pa), nearly 40 % of the initial bacterial population remained attached to the PGM coating (control, 10 %).

On the basis of our previous work (Le et al. 2013), detachment data were interpreted by evaluating the wall shear stress τ_W50%_ needed to remove 50 % of the bacteria initially attached to PS and PS + PGM. τ_W50%_ values were obtained for the IBB477 and MG1820 cells on PS and PS + PGM. On PS, τ_W50%_ reached 16.1 ± 8.4 Pa for MG1820 cells (not reached for IBB477 cells). As expected, the τ_W50%_ values on PS + PGM were substantially reduced compared to those for PS for both strains (3.8 ± 3.2 and 54.9 ± 19.3 Pa for MG1820 and IBB477 cells, respectively). The increased adhesion of the IBB477 cells to PGM coating, compared to that of the MG1820 cells, was confirmed (increase in τ_W50%_ by one order of magnitude).

### Improved high-quality draft genome sequence of *L. lactis* IBB447

**Fig. 2:**
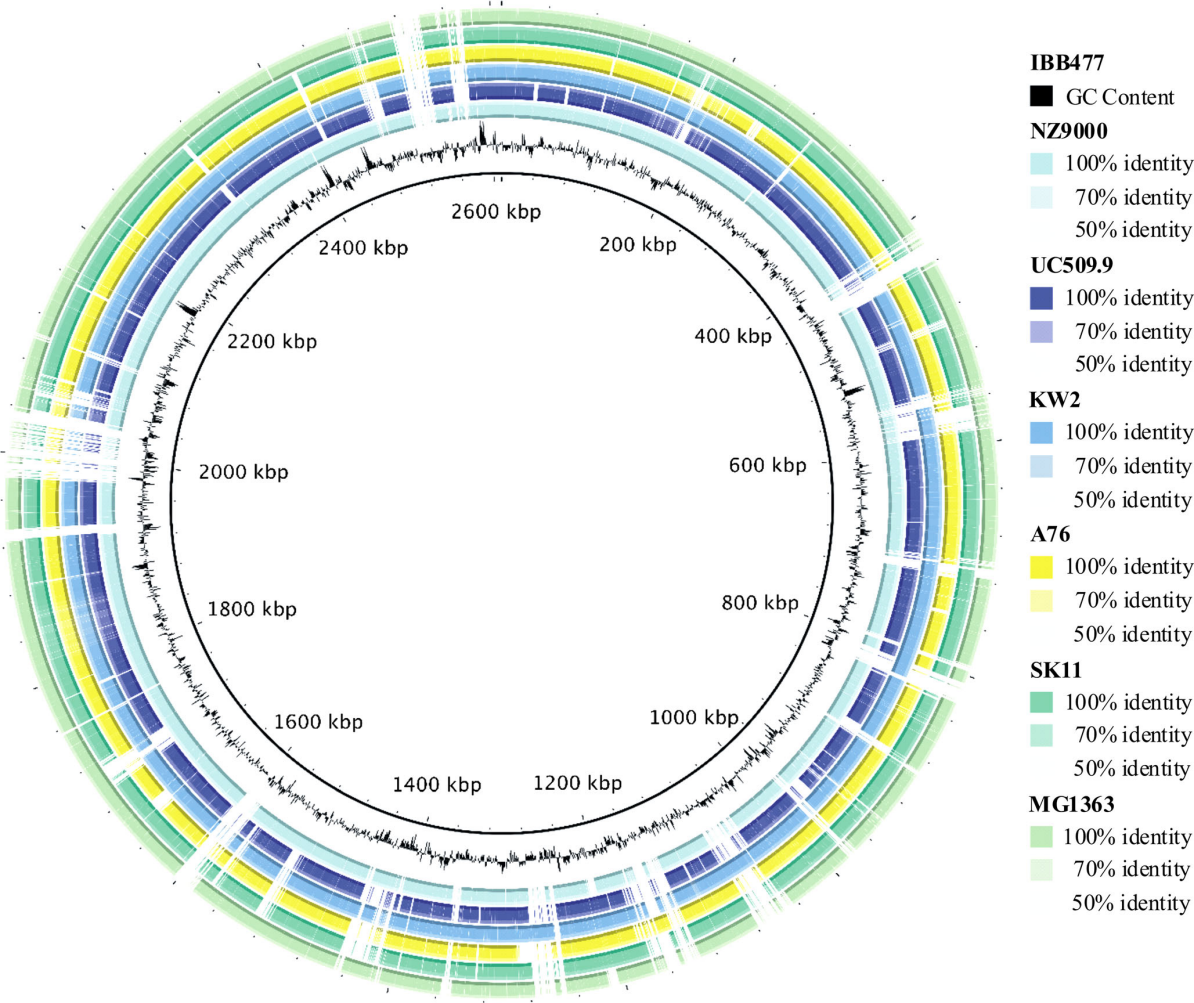
Comparison of sequence similarity between the chromosomes of *L. lactis* IBB447 (used as a query) and other sequenced *L. lactis* subsp. *cremoris* strains. Large blocks of zero similarity are due to the integration of mobile elements. Image was captured using BRIG software

To reveal genetic determinants encoding adhesive properties of the *L. lactis* IBB477 strain, the IBB477 genome has been sequenced. The genome sequence was generated by shotgun and paired-end reads by using the Roche-454 platform and Illumina sequencing technology. A total of 2.864 Mb was obtained, providing 48- and 64-fold the coverage achieved using Roche-454 and Illumina, respectively. The assembly performed with the Newbler software resulted in 125 large contigs (>500 bp) organised in seven scaffolds. Assembly of the chromosome sequence was validated with MapSolver based on the optical map produced via OpGen’s Optical Mapping System. Gap resolution by PCR and sequencing resulted in one draft and four complete sequences of plasmids. The obtained improved high-quality draft genome of *L. lactis* IBB477 consists of one scaffold composed of 35 contigs (2.6 Mb) representing the chromosomal sequence and five plasmids of different sizes: 66 kb named pIBB477a, 65 kb named pIBB477b (draft sequence), 48 kb named pIBB477c, 17 kb named pIBB477d and 12 kb named pIBB477e. The IBB477 genome has been annotated with NCBI PGAP by using GeneMarkS+ as a gene caller. A total of 2630 protein-coding genes have been identified, of which 196 show plasmid localisation. As seen in Fig. 2, the genome of *L. lactis* IBB447 is very similar to that of other *L. lactis* subsp. *cremoris* strains. The major differences (the white gaps in Fig. 2) between our strain and that of others are due to the integration of mobile elements—in each of such regions, we could identify transposase, insertion elements, or recombinases. While the total number of insertion elements identified with ISfinder database is significantly lower in the IBB447 chromosome than that in the chromosomes of other *L. lactis* subsp. *cremoris* strains (except for KW2—see Supplementary Fig. S1), only in a few cases does this translate to substantial differences between two genomes. In addition, a comparison of chromosomal organisation between *L. lactis* subsp. *cremoris* and other sequenced *L. lactis* subsp. *cremoris* strains shows the presence of inversions in the analysed sequences (Supplementary Fig. S2), which was already reported as part of a comparison study of lactococcal genomes (Kok et al. 2005). Based on a large inversion in the middle of the chromosomes (roughly from 800 to 2000 kb), *L. lactis* subsp. *cremoris* strains can be divided into two groups: one comprising KW2, UC509.9 and SK11 and another containing MG1363, NZ9000, A76 and IBB477.

### Searching for genetic determinants encoding adhesive and mucoadhesive properties of *L. lactis* IBB477

Using PSORTb, we identified 55 proteins in the chromosomal part and eight proteins on the plasmids that are either extracellular or attached to the cell wall, of which almost half (24) were annotated as ‘hypothetical’. The IBB477 genome has also been searched for the presence of putative domains involved in adhesion to mucus, extracellular matrix (ECM) or epithelial cells. As we previously described, the LPXTG-anchored mucus-binding protein (AJ89_12755), apart from the gram-positive anchor (PF00746) (no longer detected in this protein using Pfam database v. 29.0), the C-terminal anchor (PF13461) (no longer detected in this protein using Pfam database v. 29.0), and the four MucBP (PF06458) domains, contains also four partly overlapping, but larger, MUB domains (Radziwill-Bienkowska et al. 2014), which were postulated to play an important role in the adherence of LAB to the mucus layer (Boekhorst et al. 2006). Another cell wall-associated protein (AJ89_07570) with high number of different domains contains a domain of unknown function DUF285 (PF03382), C-term anchor (PF13461) ((PF13461) family has been merged into MucBP (PF06458) according to Pfam database v. 29.0) and four bacterial Ig-like domains—group 3 (Big_3) repeats (PF07523). Big_3 domains are found in a variety of bacterial surface proteins. Their function has not yet been defined, but they belong to bacterial domains with an Ig-like fold. The members of another family of bacterial Ig-like domains (Big_2) are found in bacterial and phage surface proteins. They were postulated to bind to bacterial surface carbohydrates during infection. Recently, it was demonstrated that the Hoc proteins (containing bacterial Ig-like domains) displayed on the T4 phage capsid interact with mucin, and in particular, with its highly variable glycan groups exposed to the environment (Barr et al. 2013). Pfam domain analysis of IBB477 sequence also indicated the presence of fibronectin-binding domains, namely, fibronectin-binding protein A N-terminus (FbpA) (PF05833) and streptococcal surface repeat domain (SSURE) (PF11966). The FbpA domain and the accompanying domain of unknown function DUF814 (PF05670) are also present in fibronectin-binding protein (LBA1148) of *L. acidophilus* NCFM, which have been shown to mediate adhesion to Caco2 cell line (Buck et al. 2005). SSURE is a protein fragment found to bind to fibronectin, but not to collagen or submaxillary mucin, in streptococci (Bumbaca et al. 2004). In the four proteins of IBB477, we also detected Cna protein B-type domain (Cna_B) domains (PF05738) that were previously found in *Staphylococcus aureus* collagen-binding surface protein (Deivanayagam et al. 2000). This region does not mediate collagen binding in this strain, and it is speculated that it presents the ligand-binding domain, away from the bacterial cell surface. Furthermore, in two proteins containing Cna_B domains (AJ89_05220 and AJ89_06630), the Pfam v. 29.0 analysis indicated the presence of the GramPos_pilinBB (PF16569), which is one of the major backbone units of gram-positive pili. However, there is no entire operon encoding the cell surface pili in the IBB477 genome. The von Willebrand factor type A domain (VWA) domain (PF00092), identified in one of the IBB477 proteins, has previously been demonstrated to be essential in *S. agalactiae* for PilA (pilus component) adhesion to epithelial cells (Konto-Ghiorghi et al. 2009), whereas the cell wall-binding domain of gram-positive bacteria WxL may interact with the peptidoglycan (Brinster et al. 2007) and are involved in extracellular matrix interactions (Galloway-Peña et al. 2015). Pfam analysis of the IBB477 sequence also showed the presence of an LPMO_10 domain (PF03067) that might be responsible for carbohydrate-binding activity and the motif clostridial hydrophobic W domain (ChW) (PF07538). Proteins bearing ChW repeats could be involved in adhesion or biopolymer degradation. A list of proteins suspected to be involved in adhesion or mucoadhesion is provided in Supplementary Table S1.

### Adhesion tests on *L. lactis* IBB477 strain and its mutants using microtiter plates

**Fig. 3:**
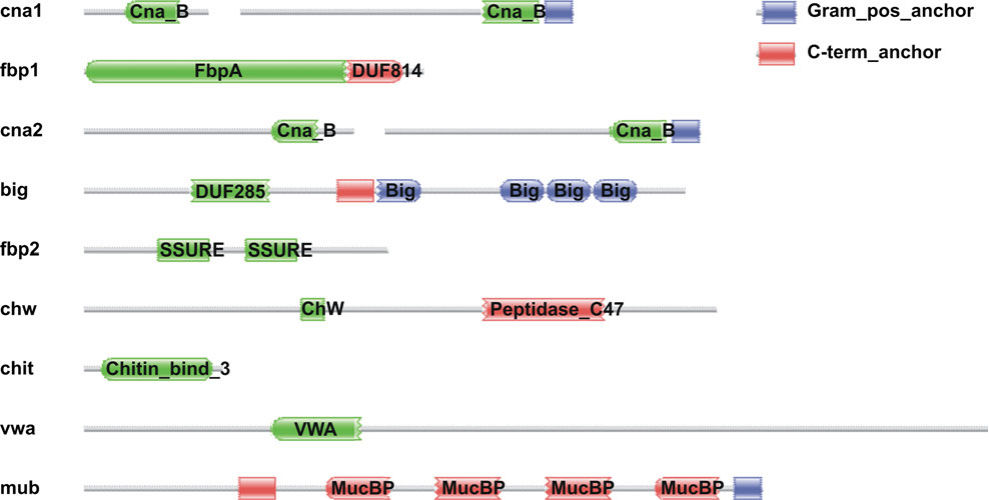
Domain modular structure of proteins encoded by chromosomal regions selected for deletion in the IBB477 strain. The putative adhesion domains were found using the Pfam database version 27.0

**Fig. 4:**
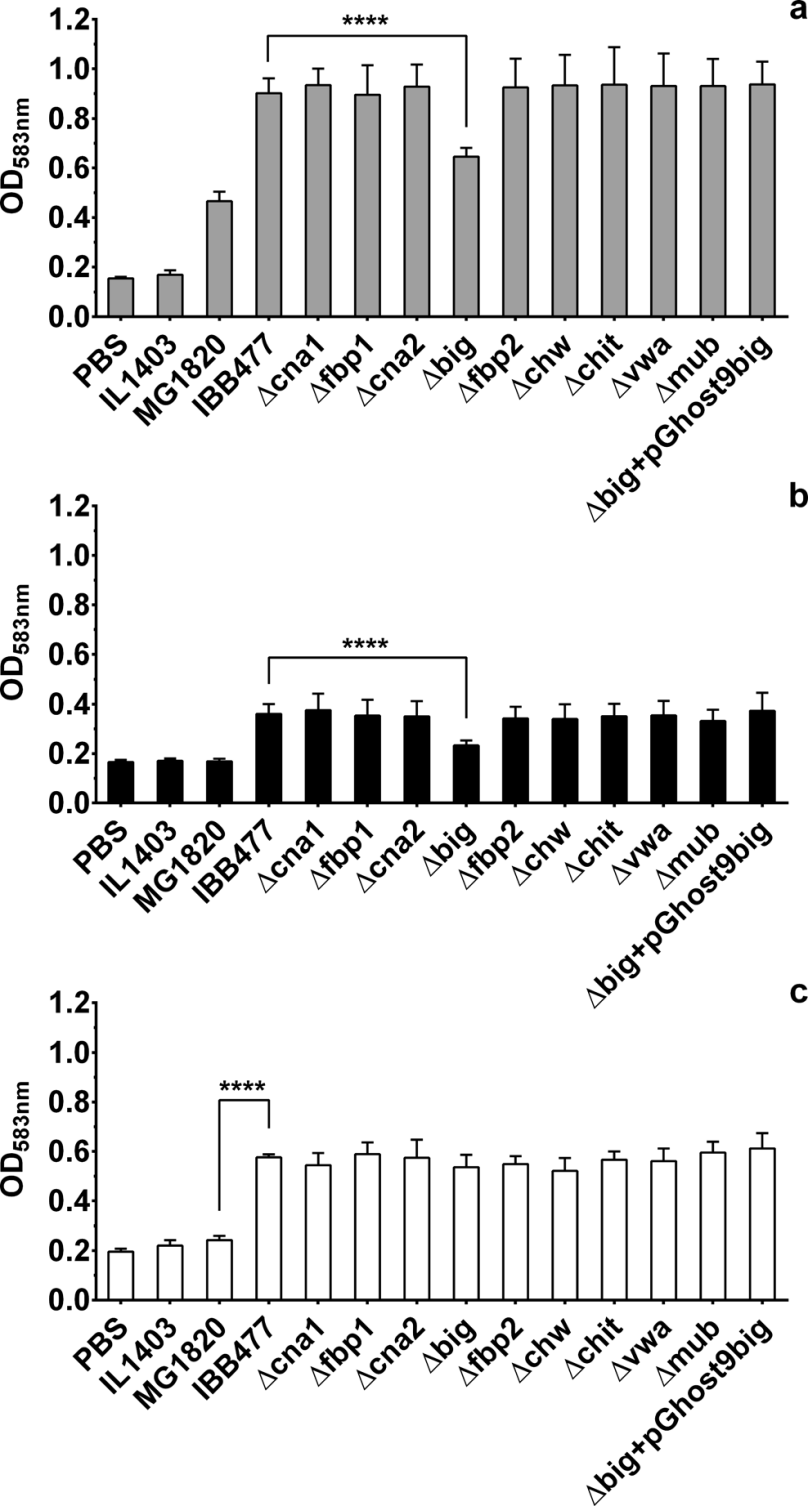
Adhesion of IBB477 and its deletion mutants in putative adhesion genes to **a** bare polystyrene (PS), **b** mucin-coated polystyrene (PS + PGM) and **c** fibronectin-coated polystyrene (PS + FN). Adhesion is expressed as optical density (OD_583nm_) of stained cells. Means ± standard deviations from three independent experiments are shown. The *p* values were calculated using Welch *t* test (**** *p* value < e^−4^). Δbig + pGhost9big-complemented Δbig mutant (containing pGhost9 with a gene encoding the AJ89_07570 protein)

Based on the obtained list of putative adhesins of IBB477 (Supplementary Table S1), nine chromosomal regions encoding proteins containing adhesion domains have been selected for deletion (Table 1). When selecting the regions, low similarity to proteins of non-adhesive *L. lactis* strains (Blastp) was taken into account apart from the presence of putative adhesion domains (Fig. 3). The adhesive properties of IBB477 deletion mutants were analyzed using adhesion tests on bare polystyrene (PS) as well as mucin-coated (PS + PGM), fibronectin-coated (PS + FN) (Fig. 4) or collagen IV-coated (PS + CN IV) (data not shown) polystyrene plates in comparison to the wild-type strain. The low-adhesive MG1820 control strain was also included for comparison. Adhesion was expressed as the optical density (OD_583nm_) of stained cells. In agreement with our results obtained for the shear stress flow chamber, adhesion to PS and PS + PGM of IBB477 strain was higher compared to the control strain MG1820. The adhesion level of IBB477 was also higher than of the *L. lactis* IL1403, which from our results appeared to be the lowest-adhesive strain. Furthermore, in the present study, about 2.4-fold higher adhesion to fibronectin-coated polystyrene of IBB477 in comparison with MG1820 was observed (*p* value <e^−4^, 95 % confidence interval (CI) = 0.32 to 0.35), whereas no differences between the analysed strains were found on collagen IV-coated plates. Among the nine IBB477 chromosomal deletion mutants, only one mutant (Δbig) in the gene AJ89_07570 encoding protein containing DUF285, C-term_anchor and four Big_3 domains adhered significantly lower than the wild-type IBB477 strain, with the *p* value < e^−4^ (95 % CI = −0.29 to −0.22) and the *p* value <e^−4^ (95 % CI = −0.15 to −0.11) to PS and PS + PGM, respectively. Δbig showed about 72 % of adherence compared with IBB477 strain to PS and ca. 65 % of adherence to PS + PGM. Deletion of the AJ89_07570 gene had no effect on the mutant’s adhesion either to PS + FN (Fig. 4) or to PS + CN IV (data not shown). Transformation of pGhost9big into IBB477Δbig, which led to the creation of the IBB477Δbig + pGhost9big strain, fully complemented the effects of AJ89_07570 deletion (Δbig), restoring the parental level of adhesion (Fig. 4). Deletion of other genes did not significantly change the adhesion level to any of the tested surfaces compared to the wild-type strain.

## Discussion

The present work first focussed on the in situ characterisation of the relationship between well-controlled hydrodynamics and *L. lactis* adhesion/mucoadhesion, by comparing two different strains: IBB477 strain, shown to exhibit in vivo persistence in the GIT of germ-free rats (Boguslawska et al. 2009), and the control strain MG1820. Bacterial cells attached to bare or pig gastric mucin (PGM)-coated polystyrene were subjected to a stepwise increase in the wall shear stress applied under laminar flow conditions. The flow chamber (inlet and outlet conditions, chamber geometry) was carefully designed to establish a fully developed laminar two-dimensional Poiseuille flow and to ensure uniform flow conditions in the observation area (Mercier-Bonin et al. 2011). Bacterial cells are exposed to hydrodynamic drag and torque, both of which increase with wall shear stress (Lorthois et al. 2001). The rate of cell removal directly correlates to their adhesive/mucoadhesive behaviour. Indeed, the importance of shear stress is increasingly being recognised. In particular, in the GIT, a dynamic environment is encountered in many compartments (e.g. shear fluctuations caused by salivary washing or intestinal peristalsis) (Jeffrey et al. 2003). The effect of shear stress on bacterial adhesion has thus been addressed in numerous works, especially when studying pathogen/host cell interactions (Konto-Ghiorghi et al. 2009; Tchesnokova et al. 2010; Thomas et al. 2002). In contrast to pathogens, the use of flow-induced shear force to probe the adhesion of beneficial bacteria like LAB has only been sporadically depicted in the literature. Likewise, data on bacterial mucoadhesion are scarce. Here, for both IBB477 and MG1820 strains under study, the presence of PGM coating substantially reduced bacterial adhesion with respect to bare polystyrene. This is in agreement with our previous results under static conditions, using the microtiter plate method (Radziwill-Bienkowska et al. 2014). The protective function generally ascribed to the mucus layer and the antiadhesive properties of mucin-based coatings (Shi et al. 2000) result from the conjunction of electrostatic, hydrophilic and steric repulsions. Despite this decrease in adhesion levels, the mucoadhesive properties of IBB477 were significantly higher than those of MG1820 (i.e. a lower fraction of detached cells from PGM coating over the entire range of wall shear stress, higher τ_W50%_ values). These trends are totally in accordance with the previous results obtained using AFM at the single-cell scale (Le et al. 2011), further confirmed by quartz crystal microbalance with dissipation monitoring (Le et al. 2012) and microtiter plate method (Radziwill-Bienkowska et al. 2014).

To the best of our knowledge, the first study on bacterial mucoadhesion under shear flow was reported for *L. lactis* subsp. *lactis* TIL448 (Le et al. 2013). This strain was previously shown to exhibit on its surface both pili and a mucus-binding protein, displaying two MucBP domains (PF06458), and which differs from the one identified by in silico analysis in the chromosome of sequenced *L. lactis* strains (Meyrand et al. 2013). Interestingly, such surface determinants are encoded by plasmid-located genes. A more important contribution of the mucus-binding protein, than pili, towards the adhesion of *L. lactis* to PGM coating was assessed under shear flow (Le et al. 2013). Accordingly, in the present work, the role of the mucus-binding protein present in *L. lactis* IBB477 was established. However, the adhesion of IBB477 cells to PGM-coated polystyrene was lower than that observed for TIL448 cells (for τ_W_ = 80 Pa, 40 and 80 % of the initial bacterial population remained attached to the PGM coating, for IBB477 and TIL448 strains), probably because of the lack of cell surface pili in the former.

To identify the molecular determinants potentially involved in the adhesion of IBB477 cells to the intestinal mucosa, the genome of this strain has been sequenced, followed by bioinformatic analysis. Cell surface-associated macromolecules are considered to play an important role in the adhesion of LAB to the GIT. Subcellular localizations of all identified proteins, as predicted by PSORTb, showed that 63 proteins were extracellular or attached to the cell wall. While the number of identified proteins might appear to be small, it is on the same order of magnitude as the experimentally identified surface proteome of *L. lactis* subsp. *cremoris* NZ9000 (Berlec et al. 2011). The choice of PSORTb v3 over other approaches or databases (such as LAB-Secretome (Zhou et al. 2010)) was dictated by the emphasis on precision (or specificity) instead of recall (or sensitivity). We strove to avoid programs that make predictions at all costs, often providing incorrect or incomplete results, which can be propagated through annotated databases and reports in the literature. LAB adhesins can bind to different targets in the intestinal mucosa: mucus, ECM and epithelial cells. Numerous extracellular, and in particular surface-associated, proteins contain many different domains (often in repeats) and domain compositions that provide information on protein functions and can be used for predicting their role in adherence. In the genome sequence of IBB477, we identified several proteins containing putative adhesion domains (Supplementary Table S1). We have detected MucBP domains (PF06458); bacterial Ig-like domains—group 3 (Big_3) (PF07523); fibronectin-binding domains: fibronectin-binding protein A N-terminus (FbpA) (PF05833) and streptococcal surface repeat domain (SSURE) (PF11966); Cna protein B-type domain (Cna_B) (PF05738); von Willebrand factor type A domain (VWA) (PF00092); WxL domain surface cell wall-binding (WxL) (PF13731); chitin-binding domain (Chitin_bind_3) (PF03067) ((PF03067) changed the name to lytic polysaccharide monooxygenase, cellulose-degrading domain (LPMO_10) according to Pfam database v. 29.0) as well as clostridial hydrophobic W domain (ChW) (PF07538).

To verify the role of putative adhesins in the adhesive IBB477 strain, functional in vitro studies were conducted considering nine chromosomal regions encoding proteins containing adhesion domains. Construction of deletion mutants was performed using an integration-excision system based on the thermosensitive plasmid pGhost9. To enable direct cloning of PCR products into pGhost9 vector and, after selection of the proper construct in *E. coli*, introduction of it into electrocompetent *L. lactis* cells, we used modified pGhost9 vector with added 3′ terminal thymidine to both ends after *Eco*RV digestion. This approach has been used for the first time for the pGhost9 vector. According to our experience, it facilitates obtaining of deletion mutants in *L. lactis* strains.

Functional studies confirmed that one of the selected genes, encoding the AJ89_07570 protein containing DUF285, C-term_anchor (recently reclassified as MucBP (PF06458) domain) and four Big_3 domains, might be involved in adhesion to abiotic surfaces as well as mucins. In general, adhesion to bare polystyrene is the result of non-specific interactions between bacteria and the abiotic surface, whereas adhesion to mucin rather results from specific interactions. In *L. lactis* adhesion to PGM, both non-specific and specific forces were observed with a higher percentage of specific adhesive events for IBB477 (20 %) compared with the control strain (5 %) (Le et al. 2011). To confirm the role of the AJ89_07570 protein in specific interactions with mucins, similar studies using AFM force spectroscopy should be performed in respect to the obtained mutant (Δbig). This protein has low similarity (BLASTP result with the highest score: 45 % query coverage and 50 % identity) to proteins of the *L. lactis* MG1363, which is a parental strain of the low-adhesive MG1820 (van Rooijen and de Vos 1990) and no homologs to proteins in IL1403 strain. Taking into account that the adhesion level of the constructed deletion mutant was still higher compared to the non-adhesive strains, other factors might also be important for adhesion of *L. lactis* IBB477. However, our studies demonstrated that none of the other selected chromosomal proteins, including the LPXTG-anchored mucus-binding protein (AJ89_12755), was related to the adhesive properties of IBB477 strain. In addition, we confirmed that IBB477 adhered better than did the low-adhesive control strain to PS and PS + PGM and we observed for the first time the higher level of adhesion of IBB477 to fibronectin-coated polystyrene surface and no differences between these strains in adhesion to collagen IV-coated plates.

Despite the high sequence similarity of the IBB477 chromosome to the genomes of other so-far sequenced *L. lactis* subsp. *cremoris* strains, IBB477 shows different adhesive properties. This might be due to the acquisition of genes related to adhesion by horizontal gene transfer, variations in the gene expression levels or the presence of plasmid-encoded genes.

In summary, we identified the putative adherence factors present in IBB477, which is, to the best of our knowledge, the first *L. lactis* strain exhibiting adhesive and mucoadhesive properties to be sequenced. Furthermore, we indicated that cell wall-associated protein (AJ89_07570) with a high number of different domains mediates adhesion to bare and mucin-coated polystyrene.

However, additional in vitro and in vivo functional studies should be performed in respect to plasmidic genes to reveal the molecular mechanisms underlying the ability of IBB477 to adhere to mucus as well as to find out whether this strain is able to persist in the GIT.

## Electronic supplementary material

ESM 1 (PDF 536 kb)
