# Supplementary material for: Adhesion of the genome-sequenced Lactococcus lactis subsp. cremoris IBB477 strain is mediated by specific molecular determinants
Source: Appl Microbiol Biotechnol. 2016 Sep 29;100(22):9605–17. doi: 10.1007/s00253-016-7813-0 (PMC5071367; doi:10.1007/s00253-016-7813-0)
Supplement: Supplementary file 1 — (PDF 536 kb) [file 253_2016_7813_MOESM1_ESM.pdf]

Supplementary material for *Applied Microbiology and Biotechnology* journal

Adhesion of the genome-sequenced *Lactococcus lactis* subsp. *cremoris* IBB477 strain is mediated by specific molecular determinants

Joanna M. Radziwill-Bienkowska, Doan T.L. Le, Pawel Szczesny, Marie-Pierre Duviau, Tamara Aleksandrzak-Piekarczyk, Pascal Loubière, Muriel Mercier-Bonin, Jacek K. Bardowski and Magdalena Kowalczyk

✉ Magdalena Kowalczyk: Institute of Biochemistry and Biophysics, Polish Academy of Sciences, Pawinskiego 5A, 02-106 Warsaw, Poland; mk@ibb.waw.pl

List of contents: Fig. S1, Fig. S2 and Table S1

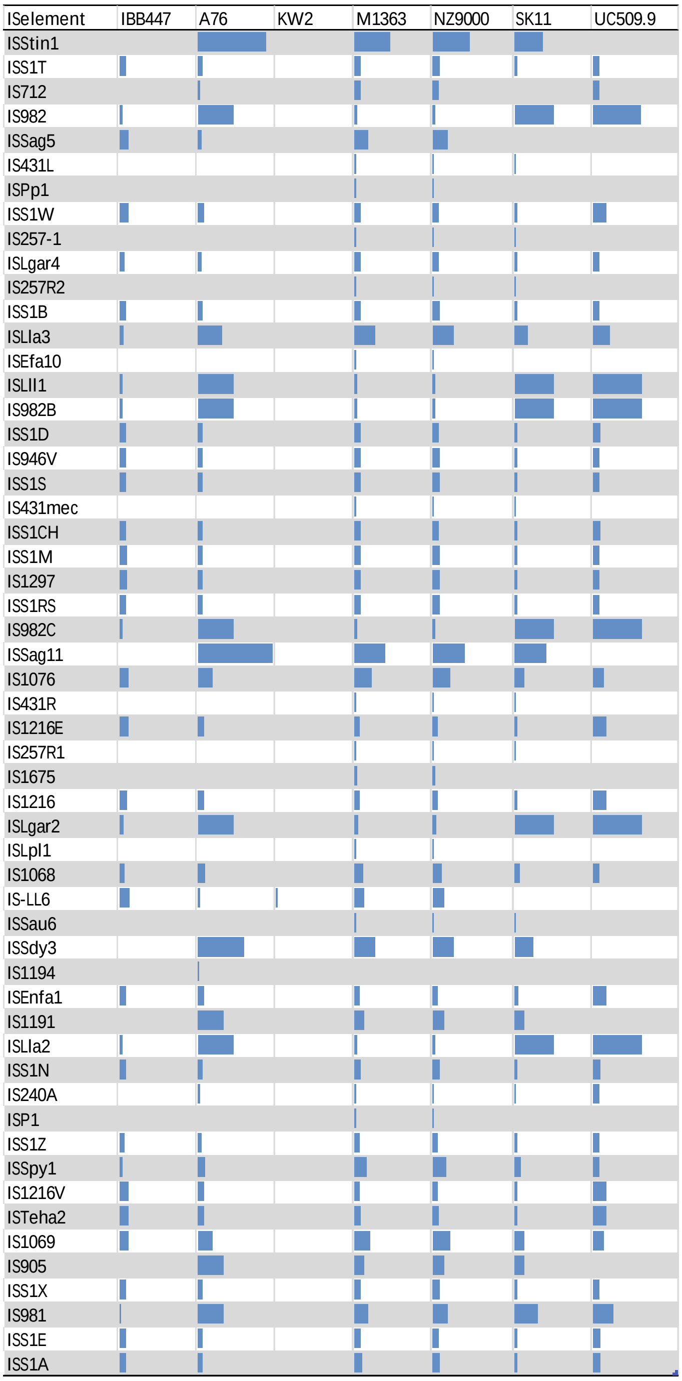

Fig. S1 Comparison of the amount of the insertion elements identified with ISfinder database in the IBB447 chromosome vs other *cremoris* genomes. Numbers were removed for clarity

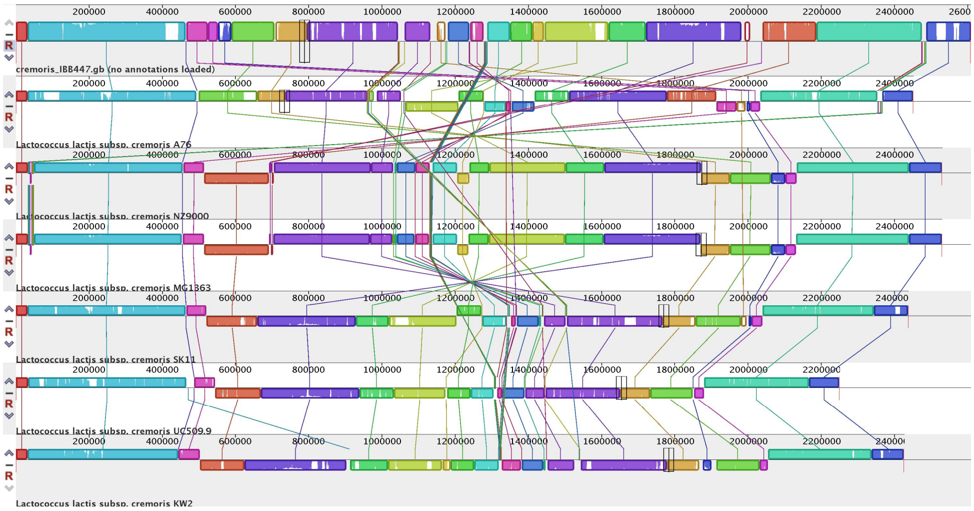

Fig. S2 Comparison of chromosomes organisation between *L. lactis* subsp. *cremoris* and other sequenced *cremoris* strains with Mauve shows the presence of large inversions in the analysed sequences

Table S1 The list of proteins from *L. lactis* IBB447 with localisation to be either extracellular or cell wall attached as well as proteins containing putative domains involved in adhesion

ID (locus|product)	Location according to PSORTb	Domain short name*	Pfam v. 27.0 No.
**CHROMOSOME**			
AJ89_00420|polysaccharide deacetylase	Extracellular		
AJ89_00850|5'-nucleotidase	Cellwall		
AJ89_01035|hypothetical protein	Extracellular	No Pfam hits	
AJ89_01065|hypothetical protein	Extracellular	No Pfam hits	
AJ89_01300|N-acetylmuramidase	Extracellular		
AJ89_01370|1,4-beta-xylanase	Extracellular		
AJ89_01490|2', 3'-cyclic nucleotide 2'-phosphodiesterase	Cellwall		
AJ89_01720|ABC transporter substrate-binding protein	Cellwall		
AJ89_02050|superoxide dismutase	Extracellular		
AJ89_02135|hypothetical protein	Extracellular	No Pfam hits	
AJ89_02360|phage minor structural protein	Extracellular		
AJ89_02390|1,4-beta-N-acetylmuramidase	Cellwall		
AJ89_02725|peptide ABC transporter substrate-binding protein	Cellwall		
AJ89_02735|N-acetylmuramidase	Extracellular		
AJ89_02850|N-acetylmuramoyl-L-alanine amidase	Extracellular		
AJ89_03260|lysozyme	Extracellular		
AJ89_03545|peptide-binding protein	Extracellular		
AJ89_04825|hypothetical protein	Extracellular	No Pfam hits	
AJ89_05045|hypothetical protein	Extracellular	No Pfam hits	
AJ89_05130|cell division protein FtsQ	Cellwall		
AJ89_05140|UDP-N-acetylglucosamine 1-carboxyvinyltransferase	Extracellular		
AJ89_05150|cell surface protein	Cellwall		
AJ89_05200|hypothetical protein	Extracellular	No Pfam hits	
AJ89_05215|hypothetical protein	Cellwall	Cna_B	PF05738

ID (locus|product)	Location according to PSORTb	Domain short name*	Pfam v. 27.0 No.
AJ89_05220|hypothetical protein	Cellwall	Cna_B,<br>Gram_pos_anchor	PF05738<br>PF00746
AJ89_05235|hypothetical protein	Extracellular	No Pfam hits	
AJ89_05390|hypothetical protein	Extracellular	No Pfam hits	
AJ89_05550|xylanase	Extracellular		
AJ89_06010|hypothetical protein	Extracellular	No Pfam hits	
AJ89_06245|lysozyme	Extracellular		
AJ89_06340|N-acetyl-muramidase	Extracellular		
AJ89_06375|hypothetical protein	Extracellular	WxL	PF13731
AJ89_06430|sugar ABC transporter substrate-binding protein	Extracellular		
AJ89_06570|dihydroorotate dehydrogenase	Cytoplasmic	FbpA<br>DUF814	PF05833<br>PF05670
AJ89_06585|hypothetical protein	Cellwall		
AJ89_06625|hypothetical protein	Unknown	Cna_B	PF05738
AJ89_06630|hypothetical protein	Cellwall	Cna_B<br>Gram_pos_anchor	PF05738<br>PF00746
AJ89_06770|sugar ABC transporter substrate-binding protein	Extracellular		
AJ89_07525|N(5)-(carboxyethyl)ornithine synthase	Extracellular		
AJ89_07570|hypothetical protein	Cellwall	DUF285<br>C-term_anchor<br>Big_3 ×4	PF03382<br>PF13461<br>PF07523
AJ89_07945|peptide-binding protein	Extracellular		
AJ89_08270|phage minor structural protein	Extracellular		
AJ89_08775|hydrolase	Extracellular		
AJ89_09345|hypothetical protein	Unknown	SSURE ×2	PF11966
AJ89_09710|hypothetical protein	Cellwall	No Pfam hits	
AJ89_09720|flagellar hook-length control protein FliK	Cellwall		
AJ89_10030|DNA-binding protein	Extracellular		
AJ89_10115|amylopullulanase	Cellwall		
AJ89_10255|hypothetical protein	Extracellular	No Pfam hits	
AJ89_10320|hypothetical protein	Unknown	ChW<br>Peptidase_C47	PF07538<br>PF05543

ID (locus|product)	Location according to PSORTb	Domain short name*	Pfam v. 27.0 No.
AJ89_10415|hypothetical protein	Extracellular		
AJ89_10555|peptide-binding protein	Extracellular		
AJ89_10735|intercellular adhesion protein	Extracellular		
AJ89_10980|surface antigen	Extracellular		
AJ89_11545|chitinase	Extracellular		
AJ89_11550|chitin-binding protein	Unknown	Chitin_bind_3	PF03067
AJ89_11995|hypothetical protein	Cytoplasmic Membrane	VWA	PF00092
AJ89_12425|competence protein ComGC	Cellwall		
AJ89_12755|internalin	Cellwall	C-term_anchor<br>MucBP ×4<br>Gram_pos_anchor	PF13461<br>PF06458<br>PF00746
AJ89_13110|hypothetical protein	Extracellular		
AJ89_13245|hypothetical protein	Extracellular	No Pfam hits	
**PLASMIDS**			
AJ89_13745|peptidase P60	Extracellular		
AJ89_13810|hypothetical protein	Extracellular	No Pfam hits	
AJ89_13930|cytochrome C oxidase subunit II	Extracellular		
AJ89_13960|N(5)-(carboxyethyl)ornithine synthase	Extracellular		
AJ89_14140|hypothetical protein	Extracellular		
AJ89_14230|peptidase S8	Cellwall		
AJ89_14545|hypothetical protein	Extracellular	No Pfam hits	
AJ89_14590|hypothetical protein	Extracellular	No Pfam hits	

* Domains are listed only in case of proteins containing at least one putative adhesive domain. Blank spaces indicate that Pfam domains are present but with no known adhesive function.
